# Systematic Immunophenotyping Reveals Sex-Specific Responses After Painful Injury in Mice

**DOI:** 10.3389/fimmu.2020.01652

**Published:** 2020-07-29

**Authors:** Vivianne L. Tawfik, Nolan A. Huck, Quentin J. Baca, Edward A. Ganio, Elena S. Haight, Anthony Culos, Sajjad Ghaemi, Thanaphong Phongpreecha, Martin S. Angst, J. David Clark, Nima Aghaeepour, Brice Gaudilliere

**Affiliations:** ^1^Department of Anesthesiology, Perioperative and Pain Medicine, Stanford University, Stanford, CA, United States; ^2^Digital Technologies Research Centre, National Research Council Canada, Toronto, ON, Canada; ^3^Department of Biomedical Data Sciences, Stanford University, Stanford, CA, United States; ^4^Department of Pathology, Stanford University, Stanford, CA, United States; ^5^Department of Pediatrics, Stanford University, Stanford, CA, United States

**Keywords:** mass cytometry, injury, inflammation, sex differences, pain, recovery

## Abstract

Many diseases display unequal prevalence between sexes. The sex-specific immune response to both injury and persistent pain remains underexplored and would inform treatment paradigms. We utilized high-dimensional mass cytometry to perform a comprehensive analysis of phenotypic and functional immune system differences between male and female mice after orthopedic injury. Multivariate modeling of innate and adaptive immune cell responses after injury using an elastic net algorithm, a regularized regression method, revealed sex-specific divergence at 12 h and 7 days after injury with a stronger immune response to injury in females. At 12 h, females upregulated STAT3 signaling in neutrophils but downregulated STAT1 and STAT6 signals in T regulatory cells, suggesting a lack of engagement of immune suppression pathways by females. Furthermore, at 7 days females upregulated MAPK pathways (p38, ERK, NFkB) in CD4T memory cells, setting up a possible heightened immune memory of painful injury. Taken together, our findings provide the first comprehensive and functional analysis of sex-differences in the immune response to painful injury.

## Introduction

**Figure 1:**
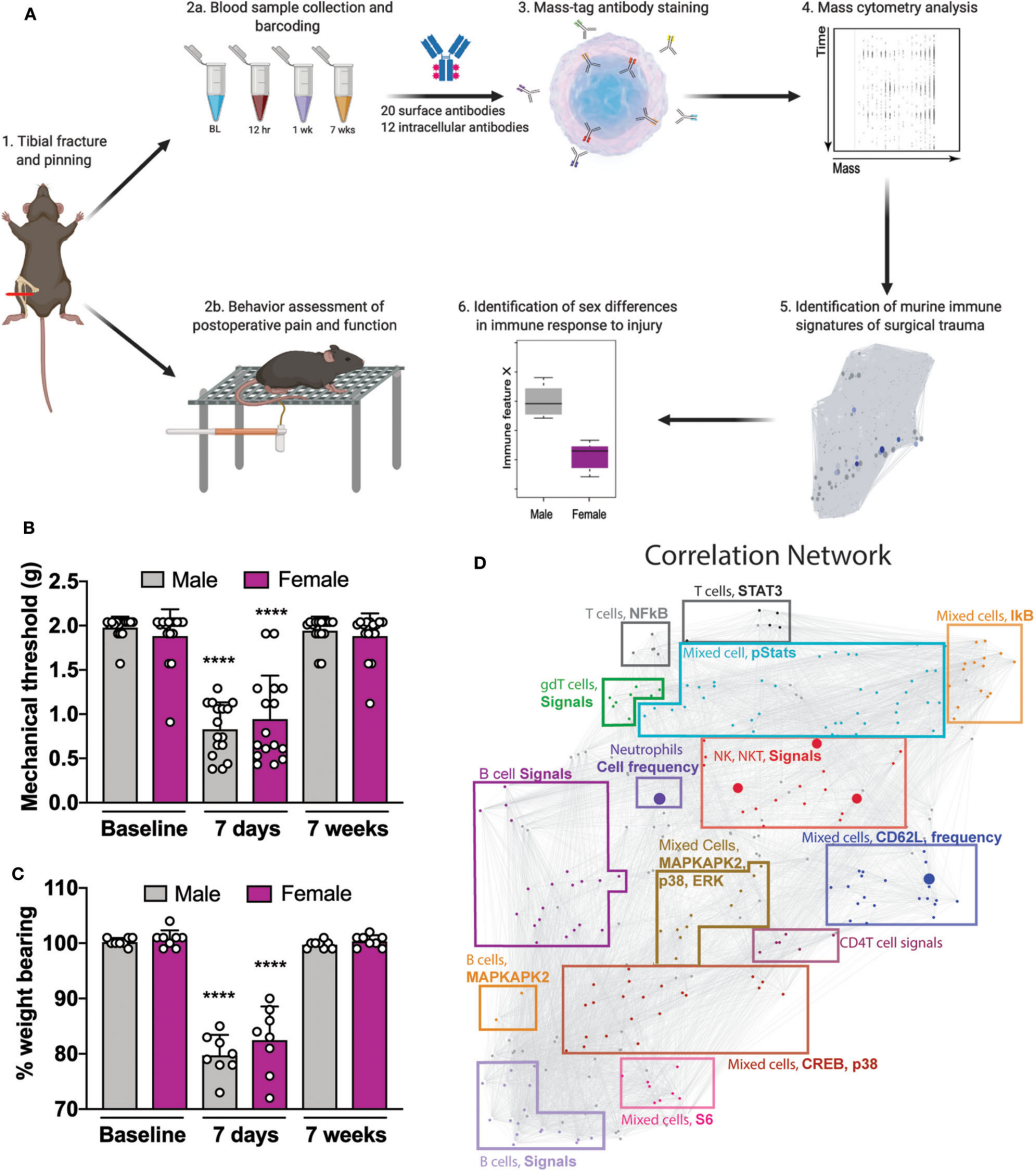
Experimental workflow and analytical approach to murine immune response after orthotrauma. **(A)** 1: Male and female mice underwent unilateral tibial fracture and pinning. 2a: A whole-blood sample was obtained at 4 time points BL, 12 h, 7 days, and 7 weeks. Samples were processed individually, barcoded for tracking purposes, and then pooled. 2b: Mice underwent behavioral assessment of postoperative pain and function through mechanical allodynia and weight bearing. 3: Aliquots were then used to quantify cell frequency and endogenous intracellular signaling activity. 4: Immune cells were stained with surface and intracellular antibodies and analyzed with mass cytometry. 5: Data obtained from mass cytometry were cross validated with an elastic net (EN) model predicting male or female at time of sampling. 6: Features that diverged between sexes were identified. **(B)** Decreased mechanical thresholds were noted 7 days after tibial fracture and pinning in male and female mice (*n* = 16 mice per group per sex, ****p < 0.0001 for 7 day threshold vs. respective sex baseline by one-way ANOVA, Tukey's post-test). **(C)** At 7 days post-injury both male and female mice placed less weight on the injured limb (*n* = 8 mice per group per sex, ****p < 0.0001 for 7 day % weight bearing vs. respective sex baseline by one-way ANOVA, Tukey's post-test). **(D)** Mass cytometry data formed a correlation network that visually segregated into 15 communities containing inter-correlated immune features that changed together during the injury recovery process. Communities are annotated based on immune feature attributes (cell type, signaling, or frequency) that are most frequently represented within each cluster. Individual data points are shown in addition to mean + SD for **(B,C)**.

Several diseases exhibit unequal prevalence between men and women. Both pre-clinical and clinical studies have suggested multiple potential contributors to this sex divergence including experiential, psychological, genetic, neurochemical, hormonal, systems-based, and sociocultural factors (1–5). Sex-divergence is particularly pronounced in inflammatory and infectious diseases, suggesting sex-specific immune responses that likely carry important treatment implications. For example, males are more susceptible to innate immune-mediated conditions such as infection (6), and women have a higher prevalence of adaptive immune-mediated autoimmune diseases (7). Inflammation is a key contributor to long-lasting pain after injury or surgery (8). It is therefore crucial to understand sex-specific differences in the post-injury immune response that may result in vulnerability to chronic pain. Here, we used a high-dimensional mass cytometry approach to quantify the distribution and intracellular signaling activities of all major innate and adaptive immune cells in male and female mice after a severe orthopedic injury (Figure 1A and Supplemental Table S1). Our goal was to identify cell-specific immunological attributes that differ between males and females after a painful injury that could guide sex-specific treatment plans for patients after injury or surgery.

## Materials and Methods

### Animals

All procedures were approved by the Stanford University Administrative Panel on Laboratory Animal Care and the Veterans Affairs Palo Alto Health Care System Institutional Animal Care and Use Committee in accordance with American Veterinary Medical Association guidelines and the International Association for the Study of Pain. Mice were housed 2–5 per cage and maintained on a 12-h light/dark cycle in a temperature-controlled environment with *ad libitum* access to food and water. Male mice weighed approximately 25 g at the start of the study, and female mice weighed ~20 g at the start of the study. All mice used in this study were wild type C57BL/6J mice (Jax stock #00664).

### Orthotrauma Model

Mice were anesthetized with isoflurane and underwent a distal tibia fracture and an intramedullary pin fixation in the right leg, as previously described (9). Briefly, to make the shaft for the bone pinning, a small hole was made in the proximal tibia and a 27 G needle was inserted down the medullary axis of the bone, and then removed. Next, the distal tibia was scored with a bone saw and fractured. To set the fracture, the 27 G needle was re-inserted into the intramedullary space, through the proximal tibia, and advanced across the fracture site to the distal portion of bone. The wound was closed with sterile staples. For behavior, mice were tested beginning 7 days after the surgery.

### Behavioral Testing

To ensure rigor in our findings, all experimenters (E.S.H., V.L.T.) performed *in vivo* behavioral testing in a blinded fashion. All testing was conducted between 7:00 am and 1:00 pm in an isolated, temperature- and light-controlled room. Mice were acclimated for 30–60 min in the testing environment within custom clear plastic cylinders (4” D) on a raised metal mesh platform (24” H). Mice were randomized by simple selection from their home cage prior to testing, and placed in a cylinder; after testing, mouse identification numbers were recorded on the data sheet.

#### Mechanical Nociception Assays

To evaluate mechanical reflexive hypersensitivity, we used a logarithmically increasing set of 8 von Frey filaments (Stoelting), ranging in gram force from 0.007 to 6.0 g. These were applied perpendicular to the plantar hindpaw with sufficient force to cause a slight bending of the filament. A positive response was characterized as a rapid withdrawal of the paw away from the stimulus filament within 4 s. Using the up-down statistical method (10), the 50% withdrawal mechanical threshold scores were calculated for each mouse and then averaged across the experimental groups.

#### Gait Analysis

Gait capture and analysis was performed using a DigiGait System (Mouse Specifics Inc., Framingham, MA). Before videos were recorded, mice were allowed to acclimate in the run chamber for 5 min and then ran at 5 and 10 cm/s for another 2–3 min to acclimate to belt movement. Once the mouse was walking steadily, the brightness, contrast and viewing frame were adjusted so that the paws could be easily seen within the captured images. The belt speed was then left constant at 15 cm/s and recording of the gait was started. Videos were recorded for as long as it took to obtain at least three segments of minimum 3 s of uninterrupted running without stopping, jumping, or paw movements off the running belt. Run videos were captured on a flat surface. Once the segments were obtained, they were post-processed and analyzed by the software provided with the DigiGait imaging system. Each segment was individually adjusted with a binary threshold adjustment tool to remove any noise and to establish well-defined paw areas. Data were manually adjusted to remove any artifacts in the paw area-plots the system included in the results. All adjustments were performed in a standardized fashion by a blinded investigator using batch analysis per DigiGait imaging system protocols. Once the data had been checked for accuracy, they were inserted into a MATLAB program to sort through the parameters, perform statistical comparisons and return an overall gait score per paw using the 42 input parameters (11) as follows:

##### Gait General Score

The percentage of parameters from an individual paw that are *not* significantly different (by unpaired two-sample *t*-test) between baseline and day 7, i.e., the value of 100% represents no significant difference from baseline in all of the measured parameters.

$$\begin{array}{l} \left(1-\frac{\#\;of\;significantly\;different\;parameters\;\left[p\;<\;0.05\right]}{F}\right)\times 100\% \end{array}$$

where *F* = 42 for the total number of measured parameters.

### Derivation of Mass Cytometry Immune Features

Whole blood samples were collected in heparin-coated syringes from *n* = 8 mice per sex per time point at baseline, 12 h, 7 days and 7 weeks by cardiac puncture. Samples from each individual mouse were processed using a standardized protocol for fixation (Smart Tube Inc.), barcoding, and antibody staining of whole-blood samples for mass cytometry analysis as previously described (12). Antibodies used for mass cytometry analysis are described in Supplemental Table S1. We based our antibody panel on our previous work in humans (12) with the goal to cover all of the major cell types in the immune system. Guided by previous clinical findings we selected intracellular markers that were altered in human surgery/trauma and shared characteristics between human and mouse. Mass cytometry data from each sample were manually gated into 21 immune cell types of interest (Supplemental Figure S1). Two categories of immune features were derived:

Cell frequency features: Cell frequencies were expressed as a percentage of gated singlets in the case of neutrophils and as a percentage of mononuclear cells (CD45+Ly6G-) in the case of all other cell types.

Endogenous functional immune features: The signal intensity of the following functional markers was simultaneously quantified per single cell: phosphorylated (p) signal transducer and activator of transcription (STAT)1, STAT3, STAT5, STAT6, phosphorylated protein(p)38 (p-p38), cAMP response element-binding protein (pCREB), and nuclear factor kappa-light-chain-enhancer of activated B cells (pNF-κB), extracellular signal-regulated kinases (ERK), mitogen-activated protein kinase-activated protein kinase 2 (MAPKAPK2), L-selectin (CD62L), plasmid ribosomal protein S6 small ribosomal subunit (S6) and total inhibitor of NF-κB (IκB). For each time point, immune features in each cell type were calculated as the difference in median signal intensity (arcsinh transformed value) of each protein compared to its baseline.

### Paw Skin Collection for Tissue Cytokines

Under isoflurane anesthesia, skin from the right (injured) hindpaw was dissected and flash frozen on dry ice. Samples were then batch processed with an *n* = 5 per time point per sex (baseline/uninjured, 7 days, 7 weeks). Protein was extracted by homogenization using a Polytron device (Brinkmann Instruments) in phosphate buffered saline (PBS) with Protease Inhibitor (Roche Applied Science) on ice. Homogenates were centrifuged at 14,000 rpm for 15 min at 4°C, and supernatants were frozen at −80°C until processing. An aliquot was subjected to the Lowry protein assay (Bio-Rad Laboratories Inc.) to normalize protein input levels to a concentration of 1 μg/μl.

### Luminex Multiplex Enzyme-Linked Immunosorbent Assay

This assay was performed by the Human Immune Monitoring Center (HIMC) at Stanford University. Mouse 38-plex Procarta kits were purchased from eBiosciences/Affymetrix/Thermo Fisher, Santa Clara, California, USA, and used according to the manufacturer's recommendations with modifications as described. Briefly, beads were added to a 96 well plate and washed in a Biotek ELx405 washer. Samples were added to the plate containing the mixed antibody-linked beads and incubated at room temperature for 1 h followed by overnight incubation at 4°C with shaking. Cold (4°C) and room temperature incubation steps were performed on an orbital shaker at 500–600 rpm. Following the overnight incubation plates were washed in a Biotek ELx405 washer and then biotinylated detection antibody added for 75 min at room temperature with shaking. Plate was washed as above, and streptavidin-PE was added. After incubation for 30 min at room temperature wash was performed as above, and reading buffer was added to the wells. Each sample was measured in duplicate. Plates were read using a Luminex 200 or a FM3D FlexMap instrument with a lower bound of 50 beads per sample per cytokine. Custom Assay Chex control beads were purchased from Radix Biosolutions, Georgetown, Texas, and were added to all wells. All data are expressed as Maximum Fluorescence Intensity (MFI) calculated against a standard curve per HIMC protocols.

### Data and Statistical Analyses

All experiments were randomized and performed by a blinded researcher. Researchers remained blinded throughout histological, biochemical, and behavioral assessments. Groups were unblinded at the end of each experiment before statistical analysis. Data are expressed as the mean + s.d. or + s.e.m. or as box-whisker plots with bars denoting maximum and minimum values, as indicated in figure legends. For behavior and cytokine studies, data were analyzed using GraphPad Prism version 8.4.1 (GraphPad Software). Data were analyzed using ordinary or repeated measures one-way or two-way analysis of variance, with a Tukey's or Sidak's *post-hoc* test, as indicated in the main text or figure captions, as appropriate.

A correlation network was generated using Spearman's correlation between every pair of functional immune features at a given timepoint. The network layout was calculated in R using the t-SNE algorithm and visualized using ggplot2. Lines between immune features represent a significant correlation (*p* < 0.05 after Bonferroni correction) between a given pair of features (13).

To build a model that identified male vs. female mice using their immunologic traits at each time point, Elastic Net analysis was implemented as previously described (14). This approach is particularly useful in the setting of data that may be highly intercorrelated (such as immune features) since this algorithm includes a penalization term that preferences for sparse models with fewer representative inter-related, but non-redundant features. The Elastic Net analysis was performed using the R package “Glmnet: Lasso and elastic-net regularized generalized linear models" (15), which is publicly available at https://cran.r-project.org/package=glmnet. The models were developed using a two-layer leave-one-out-cross-validation process. The inner layer calculates the free parameters of the EN model. The outer layer tests the model on previously unseen datapoints.

### Data Availability

Raw data are publicly available at http://flowrepository.org under experiment ID 2542. Anonymous access is provided at http://flowrepository.org/experiments/2542. Sample annotations are provided in an attachment uploaded to the repository.

## Results

### Sex-Specific Whole System Immunophenotyping After Orthopedic Trauma in Mice

To reproduce human orthotrauma, we used an established tibial fracture and intramedullary pinning model of injury that has high face and construct validity (9). Starting at 7 days post-injury, male and female mice underwent behavioral assessment of postoperative allodynia and weight bearing (Figure 1A). After injury, both male and female mice demonstrated an immediate and profound decrease in mechanical threshold after injury (Figure 1B, *F*_(15, 224)_ = 16.17, ^****^p < 0.0001 for 7 day threshold vs. respective sex baseline by one-way ANOVA, Tukey's post-test) and put less weight on the injured limb (Figure 1C, *F*_(15, 112)_ = 35.73, ^****^p < 0.0001 for 7 day % weight bearing vs. respective sex baseline by one-way ANOVA, Tukey's post-test), both of which returned to baseline by 7 weeks. Of note, unweighting provides a measure of spontaneous pain and function which is important to post-operative recovery. To further test functional deficits after orthotrauma, gait analysis was performed by having mice run on a treadmill at 15 cm/s (Supplemental Figure S2A). Using a scaled, composite score of gait dynamics per paw, we found that at 7 days post-injury, male and female mice exhibited a score that was 69.7 ± 11.8 and 73.3 ± 13.8% of baseline, indicating functional deficits in their mobility (Supplemental Figure S2B).

In this orthotrauma model, the time course and severity of pain-like behaviors were similar between males and females; however, to investigate possible sexual dimorphism in the underlying immune response, we collected blood samples for mass cytometry analysis at four time points in male and female mice: baseline (pre-injury), 12 h, 7 days and 7 weeks after injury. These time points were chosen to evaluate the preoperative, immediate postoperative, subacute and recovery phases after injury/surgery. We used a panel of 20 surface antibodies and 12 intracellular antibodies (Supplemental Table S1) to characterize cell frequency and intracellular signaling pathways in 21 immune cells spanning all major innate and adaptive compartments (Supplemental Figure S1 and Supplemental Table S2) for a total identification of 273 unique immune features. Manual gating demonstrated an increased frequency of neutrophils in males compared to females at baseline (Supplemental Figure S3, ^***^p < 0.001 by unpaired *t*-test) and as expected there was an early expansion in the neutrophil population after injury at 12 h in both sexes (Supplemental Figure S4). Intracellular signaling features in each of these cellular populations were collected and included key intracellular responses previously evaluated in patients after orthopedic surgery (12).

Visual inspection of the high dimensional dataset of 273 immune features using heat maps was performed by displaying fold change from baseline at each time point after injury, either with the data from both sexes combined or separated by sex. When intracellular signaling responses were clustered by sex (Supplemental Figure S5A, left and right columns) we identified cellular populations with visually near identical signaling responses in males and females (gray boxes, for example, neutrophils, Supplemental Figure S5B, upper panel) and others with sexually dimorphic intracellular signaling responses (orange boxes, for example, myeloid dendritic cells, Supplemental Figure S5B, lower panel). Combining sexes (Supplemental Figure S5, middle column) lost important information about sex-divergent responses to injury and highlighted the need to evaluate the immune response as a whole using a multivariate approach.

To facilitate the biological interpretation of the multivariate analysis, the high-dimensional immunological dataset was visualized as a correlation network highlighting the interconnectivity between cell-type specific immune features (Figure 1D). The network visually segregated into 15 distinct communities containing inter-correlated immune features that changed together during injury and recovery. Communities were annotated based on the cell types, cell frequency change or functional pathways most frequently represented within each module.

### An Elastic Net (EN) Model of the Immune Response Reveals Sex-Divergence at 12 h and 7 Days After Injury

**Figure 2:**
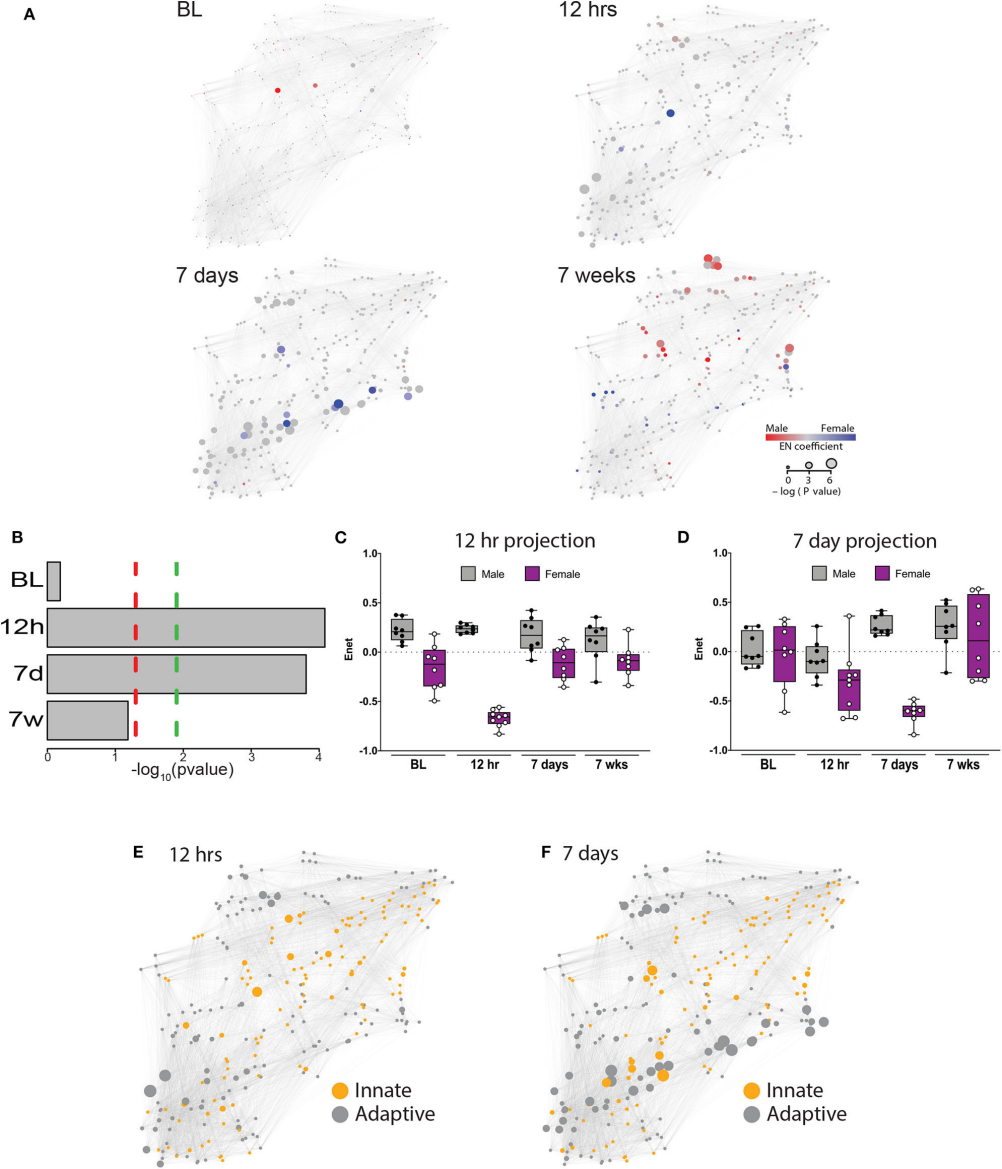
An elastic net analysis identifies a two-phase sex divergent response to injury. **(A)** Mass cytometry data were collected at 4 time points: baseline, 12 h, 7 days, and 7 weeks, and evaluated using a multivariate elastic net (EN) algorithm that characterized sex differences in the systemic response to injury and recovery. Relative size of the node corresponds to the p-value based statistical correlation between features and the intensity of red or blue color represents the absolute value of the elastic net model coefficients at that node. **(B)** The EN models were significantly different at 12 h and 7 days with (green dotted line) or without (red dotted line) Bonferroni correction for multiple comparisons (12 h, corrected *p* = 0.000329; 7 days, corrected *p* = 0.000466). Time-projected EN models demonstrate two unique sex-specific immune response periods: immediate postoperative **(C)** and subacute phase **(D)**. Immune features selected by the immediate postoperative (12 h) and subacute (7 day) elastic net models were quantified at each time point and a time-projected value was inferred for each EN model. Box plots depict range of EN model values over time, including maximum, minimum, median and interquartile range. Identification of innate and adaptive immune features demonstrates several clusters of adaptive immune features that were highly correlated at 12 h **(E)** and 7 days **(F)**.

The interconnected and highly modular nature of the data supported the use of the elastic net (EN) algorithm, a regularized regression method, to identify the immune features that were significantly different between males and females (14, 16). An EN model was created at each time point using the multivariate EN approach by inputting all the features of the analysis (cell frequencies and individual intracellular signaling changes) to identify those contributing to the model. Each model was graphically overlaid on the immune network representing the entire immunological dataset to visually display the cellular and intracellular features differing between males and females at a given time point (Figure 2A, -log(p value) ∝ increasing size of node; EN coefficient ∝ red -> blue color). At baseline and 7 weeks post-injury, the EN analysis did not differentiate samples between male and female mice, suggesting that overall measured immune responses were similar in males and females before injury and after the recovery period (Figure 2B). In contrast, at 12 h and 7 days after injury, robust EN models were identified that differentiated male and female samples (Figure 2B, red line *p* value = 0.05, green line Bonferroni adjusted *p* value = 0.05), suggesting two distinct sex-dependent phases of the systemic immune response to injury. The 12 h model was built on 65 features (Supplemental Table S3) and had a Bonferroni corrected p-value of 0.000329 (Figure 2B). The 7-day model used 52 features (Supplemental Table S4) and had a Bonferroni corrected p-value of 0.000466 (Figure 2B). At 7 days after injury, the majority of these features were higher in females compared to males, suggesting an exacerbated immune response to injury in females compared to males (Supplemental Table S4, negative EN coefficients denote features that were up in females compared to males).

To estimate the magnitude of sex-differences in the immune response after painful injury, the 12 h and 7d EN models were projected onto the immunological dataset from all time points. Results from these projections revealed profound differences between males and females that peaked at 12 h (males: median 0.24, range 0.18–0.30; females: median −0.66, range −0.83 to −0.56, Figure 2C) and 7d (males: median 0.22, range 0.16–0.41; females: median −0.60, range −0.84 to −0.48, Figure 2D). Interestingly, when projected to all time points, the 12 h EN model revealed sex-differences present at all time points, including at baseline, while differences observed using the 7d EN model were confined to the 7d time point.

The EN method allowed a system-level analysis of differential immune responses between males and females after injury, anchored by a statistically-stringent multivariate model, revealing a chronology of interrelated immune events that characterized the sexually dimorphic immune response to injury and recovery. As a result of known differences in innate and adaptive immunity between males and females (17), we classified features by innate or adaptive cell type in each of the EN models with significant predictive power (Figure 2E: 12 h; Figure 2F: 7 days). As shown in Figure 2E, there are at least 2 independent clusters of adaptive features (gray) that are highly correlated and one central highly correlated innate cluster (orange). Strikingly, at 7 days after injury there were several clusters of adaptive immune features that were highly correlated and had significant p values (Figure 2F, large gray nodes).

### Females Downregulate Signaling in T Regulatory Cells and Upregulate Signaling in CD4T Memory Cells After Injury

**Figure 3:**
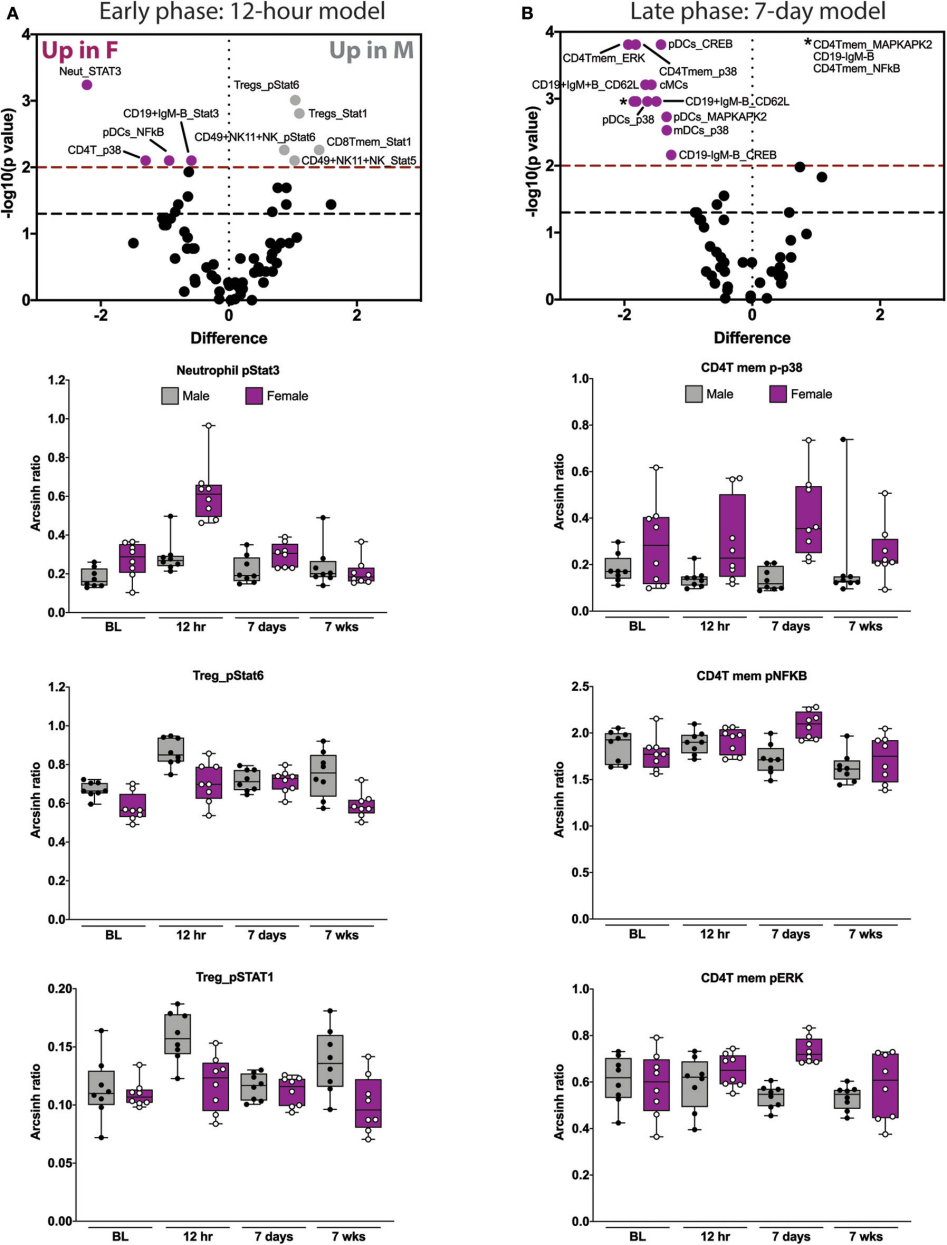
Elastic net components reveal time- and sex-specific alterations in innate and adaptive immune responses after orthotrauma. **(A)** Early (immediate postoperative) and **(B)** late (subacute) phase models illustrate all features that contribute to the model (i.e., have non-zero EN coefficient). Three highly informative EN components are shown below each model. In the early model, STAT signaling in neutrophils and Tregs are most informative. In the late model, CD4Tmem MAPK signaling components are most informative. Box plots depict range of arcsinh ratio values over time, including maximum, minimum, median and interquartile range. cMCs, classical monocytes; pDCs, plasmacytoid dendritic cells.

In response to injury the immune system exhibits alterations in intracellular signaling, and functional consequences of these changes provide a feedback loop essential to the injury response. Thus, we next evaluated the individual features that contributed to the 12 h and 7-day models in order to determine which cell types and intracellular signaling pathways were the most informative components of the sex-specific phase-specific response to injury. As shown in Figure 3A (12 hrs) and Figure 3B (7 days), a limited number of cell types showed several sex-divergent intracellular responses. Additionally, certain intracellular signaling proteins, such as STATs, were significantly altered in multiple cell types. Taken together, these findings suggest that even when behavioral readouts of pain are similar, there is a sex-specific response to injury that diverges with preference for certain specific cell types and signaling cascades.

We next examined the three most informative components of each of the time-based EN models. The most informative features of the 12 hrs EN model consisted of STAT signaling responses. Compared to males, females demonstrated an increase in neutrophil pSTAT3 and a decrease in pSTAT1 and pSTAT6 in Tregs (Figure 3A and Supplemental Figure S6). The most informative components of the 7-day EN model consisted of MAP kinase signaling responses in CD4T memory cells. Compared to males, females demonstrated an increase in p-p38, pNFκB and pERK (Figure 3B and Supplemental Figure S7) in CD4T memory cells.

There are several features that were conserved between sexes including canonical injury responses. Specifically we observed (1) a decrease in neutrophil CD62L at 12 h in both sexes suggesting rapid egress of neutrophils from blood to the tissue site of injury; (2) an increase in classical monocyte CD62L at 12 h and then subsequent decrease at 7 days suggesting a delayed transmigration into tissues and (3) an increase in classical monocyte pSTAT3 at 12 h suggesting immediate activation of these cells after injury (Supplemental Figure S8).

### Females Exhibit Increased Levels of Several Proinflammatory Cytokines in the Injured Hind Paw Compared to Males

**Figure 4:**
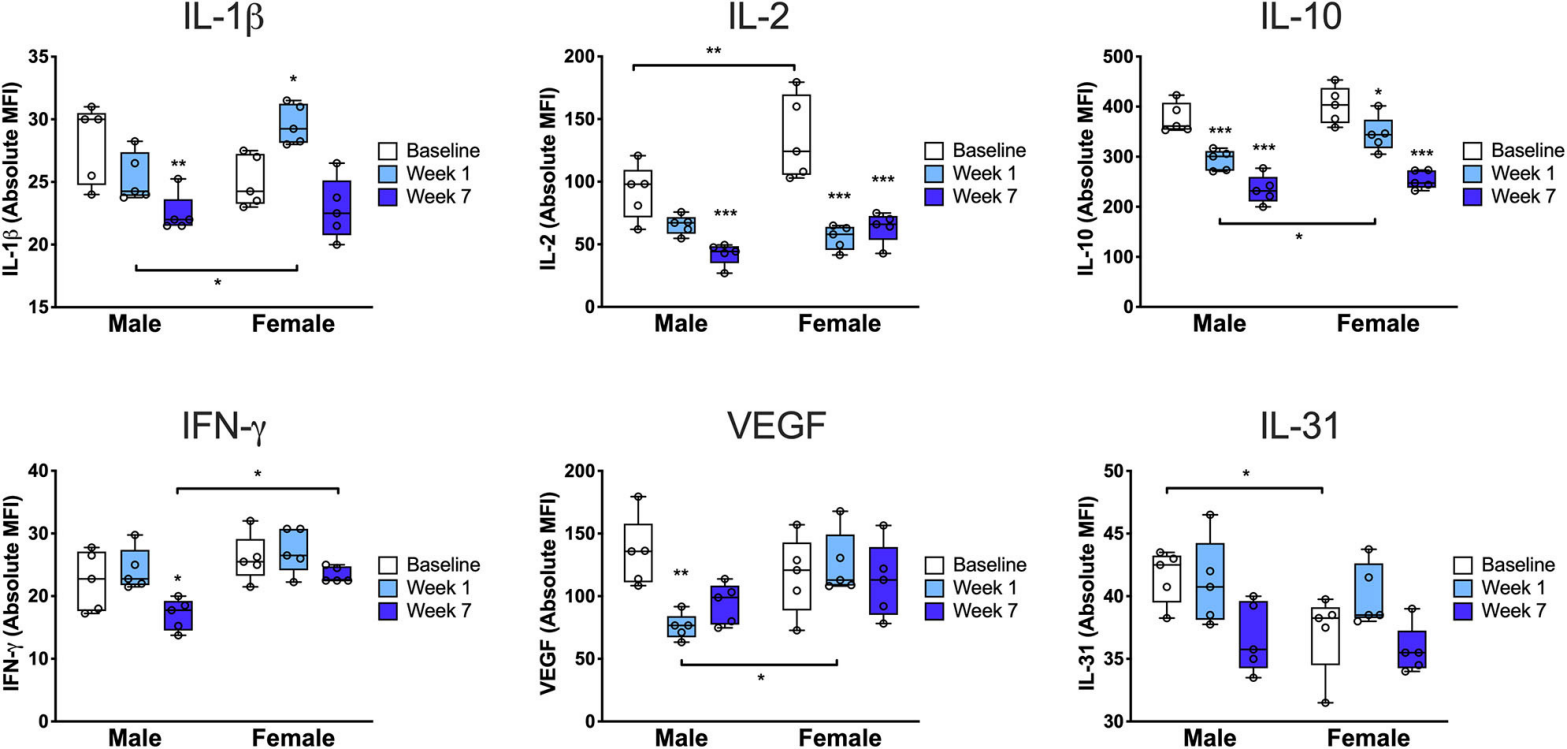
Cytokine profile from the injured limb demonstrates sex differences in the tissue immune response. Paw skin protein was subjected to multiplex enzyme-linked immunosorbent assay to determine the levels of key cytokines at baseline, 7 days and 7 weeks after injury. At baseline, females exhibited increased levels of IL-31 and IL-2, a Treg differentiation factor, followed by a significant drop in IL-2 at 7 days and 7 weeks post-injury. Compared to males at 7 days, females exhibited higher levels of the proinflammatory cytokine IL-1β and the pro-angiogenic/endothelial permeability factor VEGF as well as higher levels of the anti-inflammatory cytokine, IL-10. Compared to males at 7 weeks, females had higher levels of tissue IFN-γ (*n* = 5 mice per group per sex, *p < 0.05, **p < 0.01, ***p < 0.001 vs. respective sex baseline or respective time point male vs. female, as indicated in the figure, by two-way ANOVA, Sidak's *post-test*).

In order to more fully characterize the sex-specific immune response to injury, we next performed multiplex analysis of a panel of 38 cytokines and chemokines using protein isolated from the injured limb compared to the uninjured/baseline condition. We identified six proteins that were differentially regulated between males and females (Figure 4). At baseline there were only two cytokines that were differentially expressed by sex: IL-2 and IL-31. Interestingly, IL-2, a Treg differentiation factor, was increased in females compared to males at baseline (^*^p < 0.01 vs. males) but then decreased significantly in females by 7 days (^***^p < 0.001 vs. baseline). At 7 days females exhibited higher levels of the proinflammatory cytokine IL-1β, both compared to males (^*^p < 0.05 vs. males) and compared to baseline (^*^p < 0.05 vs. baseline). Furthermore, males downregulated the pro-angiogenic/endothelial permeability factor VEGF at 7 days (^**^p < 0.01 vs. baseline) while females did not (^*^p < 0.05 vs. males). We also found that at 7 weeks, males downregulated IFN-γ compared to baseline (^*^p < 0.05 vs. baseline) and to females (^*^p < 0.05 vs. females). In addition, we identified twelve proteins with changes that were either sex-conserved or only changed in one sex (Supplemental Figure S9). Males and females both exhibited increased levels of IL-23, eotaxin, RANTES, and IP-10 at 7 days and decreased levels of IL-9, IL-27, and GroA at 7 days and 7 weeks.

## Discussion

In this preclinical study, we used a single-cell immune profiling approach with mass cytometry combined with machine learning algorithms to analyze our high-dimensional immune dataset in a clinically informed mouse model of orthotrauma to determine whether females and males differ in their immune adaptations to a painful injury. Overall, we have shown similar development of allodynia and unweighting in males and females after orthotrauma but a divergence of underlying immune responses to this injury. This is particularly important because while both men and women may experience pain after injury or surgery, sex-specific treatments may be warranted. We have identified a unique immune profile in females: an increased neutrophil and dampened Treg response in the acute post-injury period, and heightened CD4T memory cell mitogen-activated protein kinase (MAPK) response in the subacute post-injury period. Tregs primarily function to inactivate traditional T cells to limit autoimmune reactions (18, 19). Our findings suggest that Tregs in females are less activated in the immediate postoperative period, perhaps resulting in a stronger early adaptive immune response to injury. As crucial priming cells, CD4T memory cells home to an area of injury and become activated after presentation of antigen (20). Within CD4T memory cells, we observed phosphorylation of MAPK pathway components (p38, NFkB and ERK) implying greater activation and more efficient secondary immune response (21). These findings have implications for recovery from injury and subsequent susceptibility to chronic pain and autoimmune dysfunction, particularly in light of the increased incidence of these conditions among females (6).

The immune response to injury is complex and involves a broad range of interdependent cell types and signaling pathways. To our knowledge, this is the first time cytometry by time-of-flight (CyTOF) mass spectrometry has been used to track the whole system immune response to injury in male and female mice. Such high parameter data requires rigorous computational methods to be analyzed effectively given the high risk of false positives. We have previously applied the EN model, a regularized regression method, to the analysis of human CyTOF datasets (16, 22). In the current study, application of the EN method revealed that at baseline and 7 weeks, the overall immune system did not differ between males and females, however, at 12 h and 7 days, a small subset of features was predictive of the sex-specific response.

Baseline differences in immune cell populations between sexes have been previously reported in the peritoneal and pleural cavities (23) as well as peripheral blood (3). Using a model of mesenteric or renal ischemia/reperfusion, Madalli et al. (24) demonstrated increased neutrophils in blood and increased recruitment of these innate immune cells into tissues in males compared to females. While we did identify an increase in baseline neutrophil frequency in males compared to females, one of the most important features of the 12 hrs model was an increase in neutrophil pSTAT3 in females. STAT3 is a transcription factor with varied roles including orchestrating proliferative and migratory functions of neutrophils but also limiting these responses in order to avoid destructive inflammation (25). Increases in pSTAT3 in neutrophils likely occur via IL-6 binding to its cognate receptor, suggesting that such proinflammatory cytokines may exist in higher abundance in females after injury. Neutrophil numbers and degranulation have further been shown to increase in response to estrogens and their release of proinflammatory mediators suppressed by androgens (17, 26). Neutrophils may contribute more significantly to early pain and injury responses in females compared to males. We have also recently demonstrated that STAT3 signaling is increased in innate immune subsets after stroke in humans (22). This suggests some immune system commonalities between disparate injuries, though our human sample size was not large enough to discern sex-specificity.

The higher prevalence of autoimmune diseases in women compared to men suggests an overactive adaptive immune response to injury or infection (7). For example, women mount a stronger adaptive immune response to vaccination, displaying an equivalent antibody response with half the dose of influenza vaccine compared to men (27). Our current data support these findings in a novel fashion, highlighting that the adaptive immune response to injury in females is more pronounced than in males. In our EN model, we found several highly correlated adaptive immune features at both 12 h and 7 days (Figures 2E,F) including both B and T cell features. Additionally, the most informative components of the 7 day model comprised almost exclusively male-female differences in adaptive immune cells and in every case the individual features were increased in females. Remarkably, JAK/STAT signaling responses in Tregs—a cell type that suppresses adaptive immune cell responses—were decreased in females compared to males. Importantly, we also demonstrated a significant decrease in IL-2, a Treg differentiation factor, in the injured limb of females at 7 days and 7 weeks post-injury. These findings are consistent with a more robust adaptive immune response to injury observed in females lacking the crucial Treg brake. While we did not observe any behavioral differences between male and female mice in our assays it is clear that the immune mechanisms underlying the injury response may predispose women more strongly to subsequent chronic pain conditions.

Almost all painful disorders disproportionately affect women. For example, the injury and autoimmune-related condition, complex regional pain syndrome (CRPS), has a 4:1 female: male distribution (28, 29), and epidemiological data suggests a 5.5% excess female prevalence for common pain syndromes such as back pain, widespread body pain, migraine, neuropathic pain, musculoskeletal pain and osteoarthritis pain (1). Several studies have suggested a contribution of T cells to pain in both mouse and human studies (30). For example, in a group of 14 patients with post-injury CRPS, Russo et al. (31) demonstrated an expansion of CD3+ T cells as well as increased pNFkB in both CD4+ and CD8+ T memory cells in peripheral blood by mass cytometry. They did not have a large enough cohort to discern the sex-specificity of these immune responses, however, ten of the fourteen recruited patients were female. In mouse models, it has been demonstrated that T cells infiltrate the central nervous system (CNS) (32), and Rag1^−/−^ mice exhibit reduced pain sensitivity after peripheral nerve injury (33). The attribution of this latter finding to T cell deficiency in Rag1^−/−^ mice warrants further study given that these mice lack both mature B and T cells from birth and compensatory changes are likely (34). Interestingly, several studies summarized comprehensively by Laumet et al. (30) have used T cell reconstitution as an approach to more specifically implicate T cells and their subsets in pain responses. One such study demonstrated that adoptive transfer of CD8T cells increased chemotherapy-induced neuropathic pain while adoptive transfer of Tregs suppressed neuropathic pain in mice (35). Unfortunately, the majority of studies were either performed in male mice or the sex of the mice was not listed, and therefore the sex specificity of such findings remains unclear. One prior study has investigated the contribution of T cells to female post-injury pain behaviors (3). Based on data that female T cells may bias toward the production of interferon-γ (36), they showed that the PPARγ agonist pioglitazone reverses nerve injury-induced pain behaviors, presumably through suppression of interferon-γ. Our own findings are consistent with this potential mechanism as we found that males downregulated IFN-γ expression in the injured limb at 7 weeks while females did not. IFN-γ is most known as a response to viral and bacterial infections; however, it has also been found to contribute to autoimmune and autoinflammatory diseases which has important implications for such female-predominant conditions (37). Finally, Rosen et al. (38) used adoptive transfer of CD4T cells in *nude* mice to show that these cells mediate sex differences in pain sensitivity and morphine analgesia. Taken together, these studies support our current work establishing subsets of T cells as key drivers of sex differences in the injury response.

Mass cytometry provides an unbiased approach to conduct a comprehensive analysis of phenotypic and functional immune system sex differences after injury; however, it provides mainly correlative data. As a result, another plausible interpretation of our findings is that the system-wide immune response to injury is largely independent and uncoupled to pain responses in this model. This would suggest that there are sex-specific immune profiles of injury alone, and that the underlying mechanisms of injury-induced pain may require evaluation of CNS, and not peripheral, immune responses. Future studies are underway to further explore the specific causative roles of early neutrophil activation and decreased Treg JAK/STAT signaling to the female susceptibility to chronic pain and autoimmunity as well as the downstream consequences of overactivation of CD4T memory MAPK signaling pathways. Our findings further highlight the importance of using both male and female mice in preclinical studies (1) and clearly indicate that combining data from sexes risks overlooking crucial, and potentially actionable, findings. Taken together, through the use of a unique, longitudinal, high-dimensional analysis, we have discovered that despite similar behavioral responses to injury, the whole system immune response to injury is sexually dimorphic. These broadly applicable results will open new avenues for the exploration of sex-specific treatment paradigms for patients with chronic pain.

## Data Availability Statement

The datasets presented in this study can be found in online repositories. The names of the repository/repositories and accession number(s) can be found below: http://flowrepository.org/experiments/2542.

## Ethics Statement

The animal study was reviewed and approved by Stanford University Administrative Panel on Laboratory Animal Care and the Veterans Affairs Palo Alto Health Care System Institutional Animal Care and Use Committee in accordance with American Veterinary Medical Association guidelines and the International Association for the Study of Pain.

## Author Contributions

VT and EH designed and performed behavioral studies. TP performed MATLAB analyses. EG and BG performed mass cytometry data collection and curation. NA performed or supervised all statistical modeling and analysis with assistance from AC and SG. VT, NH, and QB interpreted mass cytometry data. VT, NA, and BG wrote the manuscript. All authors contributed to editing of the manuscript and approved the final version. VT, NA, BG, and JC supervised all experiments. The project was conceived by VT, BG, and JC.

## Conflict of Interest

The authors declare that the research was conducted in the absence of any commercial or financial relationships that could be construed as a potential conflict of interest.
