# Supplementary material for: Systematic Immunophenotyping Reveals Sex-Specific Responses After Painful Injury in Mice
Source: Front Immunol. 2020 Jul 29;11:1652. doi: 10.3389/fimmu.2020.01652 (PMC7403191; doi:10.3389/fimmu.2020.01652)
Supplement: Supplementary file 1 [file Table_1.PDF]

Supplemental Table S1. Antibody Panel used for Mass Cytometry Analysis

Antigen	Atomic Symbol	Atomic Mass	Clone	Supplier	Comment
CD15	Y	89	Polyclonal	NSJ Bioreagents	Phenotype
Barcode 1	Pd	102		Trace Sciences	Barcode
Barcode 2	Pd	104		Trace Sciences	Barcode
Barcode 3	Pd	105		Trace Sciences	Barcode
Barcode 4	Pd	106		Trace Sciences	Barcode
Barcode 5	Pd	108		Trace Sciences	Barcode
Barcode 6	Pd	110		Trace Sciences	Barcode
Ter-119	In	113	TER-119	Biolegend	Phenotype
CD45	In	115	30-F11	Biolegend	Phenotype
CD24*	La	139	M1/69	Biolegend	Phenotype
CD27*	Ce	140	LG.3A10	Invitrogen	Phenotype
Ly6G	Pr	141	1A8	Fluidigm	Phenotype
CD11c	Nd	142	N418	Fluidigm	Phenotype
CD11b*	Nd	143	M1/70	Fluidigm	Phenotype
CD115*	Nd	144	AFS98	Fluidigm	Phenotype
CD4	Nd	145	RM4-5	Fluidigm	Phenotype
CD8a	Nd	146	53-6.7	Fluidigm	Phenotype
PDCA-1	Sm	147	129C1	Biolegend	Phenotype
pCreb	Nd	148	87G3	Cell Signaling Technologies	Function
CD19	Sm	149	6D5	Fluidigm	Phenotype
pStat5	Nd	150	C11C5	Cell Signaling Technologies	Function
p-p38	Eu	151	36/p38	BD	Function
CD3e	Sm	152	145-2C11	Fluidigm	Phenotype
pStat1	Eu	153	14/P-STAT1	Cell Signaling Technologies	Function
pStat3	Sm	154	M9C6	Cell Signaling Technologies	Function
pS6	Gd	155	D57.2.2E	Cell Signaling Technologies	Function
Foxp3	Gd	156	NRRF-30	eBioscience	Phenotype
CD25	Gd	157	3C7	Biolegend	Phenotype
CD16	Gd	158	2.4G2	BD	Phenotype
TCRγδ	Tb	159	GL3	Fluidigm	Phenotype
CD62L	Gd	160	MEL-14	Fluidigm	Function
cParp*	Dy	161	Polyclonal	Invitrogen	Function

Ly6C	Dy	162	HK1.4	Fluidigm	Phenotype
Tbet*	Dy	163	4B10	eBioscience	Function
IκB	Dy	164	L35A5	Cell Signaling Technologies	Function
NK1.1	Ho	165	PK136	Fluidigm	Phenotype
pNFκB	Er	166	K10-895.12.50	BD	Function
pErk1/2	Er	167	D13.14.4E	Cell Signaling Technologies	Function
pMapkapk2	Er	168	27B7	Cell Signaling Technologies	Function
IgM	Er	169	R6-60.2	BD	Phenotype
CD49b	Er	170	HMa2	Fluidigm	Phenotype
CD44	Yb	171	IM7	Fluidigm	Phenotype
pStat6	Yb	172	46H1L12	Invitrogen	Function
CD38*	Yb	173	90	Biolegend	Function
MHCII	Yb	174	M5/114.15.2	Fluidigm	Phenotype
CD127*	Lu	175	A7R34	Fluidigm	Phenotype
B220	Lu	176	RA3-6B2	Fluidigm	Phenotype
DNA	Ir	191		Fluidigm	DNA
DNA	Ir	193		Fluidigm	DNA

*Not included in final analysis
