# Supplementary material for: Systematic Immunophenotyping Reveals Sex-Specific Responses After Painful Injury in Mice
Source: Front Immunol. 2020 Jul 29;11:1652. doi: 10.3389/fimmu.2020.01652 (PMC7403191; doi:10.3389/fimmu.2020.01652)
Supplement: Supplementary file 2 [file Table_2.PDF]

Supplemental Table S2: Cell population definitions

Population	Abbreviation	Markers
Neutrophils	Neutrophils, Gr	CD45+ Ly6G+
Natural Killer T-cells	NKT	CD45+ Ly6G- CD3+ CD19- CD49b+
CD4+ T-cells	CD4T	CD45+ Ly6G- CD3+ CD19- CD4+
CD8+ T-cells	CD8T	CD45+ Ly6G- CD3+ CD19- CD8a+
CD4+ Naïve T-cells	CD4Tnaive	CD45+ Ly6G- CD3+ CD19- CD4+ CD44-
CD8+ Naïve T-cells	CD8Tnaive	CD45+ Ly6G- CD3+ CD19- CD8a+ CD44-
CD4+ Memory T-cells	CD4Tmem	CD45+ Ly6G- CD3+ CD19- CD4+ CD44+
CD8+ Memory T-cells	CD8Tmem	CD45+ Ly6G- CD3+ CD19- CD8a+ CD44+
Regulatory T-cells	Treg	CD45+ Ly6G- CD3+ CD19- CD4+ CD25+ Foxp3+
Gamma-delta T-cells	gdT	CD45+ Ly6G- CD3+ CD19- CD4- CD8a- TCRγδ+
B220+ Natural Killer Cells	B220+NK	CD45+ Ly6G- CD3- CD19- CD49b+ B220+
CD49+ NK1.1- Natural Killer Cells	CD49+NK11-NK	CD45+ Ly6G- CD3- CD19- CD49b+ NK1.1-
CD49+ NK1.1+ Natural Killer Cells	CD49+NK11+NK	CD45+ Ly6G- CD3- CD19- CD49b+ NK1.1+
CD19+ IgM+ B-cells	CD19+IgM+B	CD45+ Ly6G- CD3- B220+ CD19+ IgM+
CD19+ IgM- B-cells	CD19+IgM-B	CD45+ Ly6G- CD3- B220+ CD19+ IgM-
CD19- IgM- B-cells	CD19-IgM-B	CD45+ Ly6G- CD3- B220+ CD19- IgM-
Classical Monocytes	cMCs	CD45+ Ly6G- CD3- CD19- CD49b- Ly6C+
Non-classical Monocytes	ncMCs	CD45+ Ly6G- CD3- CD19- CD49b- Ly6C- CD16+
Intermediate Monocytes	intMCs	CD45+ Ly6G- CD3- CD19- CD49b- Ly6C+ CD16+
Conventional (Myeloid) Dendritic Cells	mDCs	CD45+ Ly6G- CD3- CD19- CD49b- Ly6C- CD16- CD11c+ MHCII+
Plasmacytoid Dendritic Cells	pDCs	CD45+ Ly6G- CD3- CD19- CD49b- Ly6C- CD16- PDCA1+ MHCII+
