# Supplementary material for: Systematic Immunophenotyping Reveals Sex-Specific Responses After Painful Injury in Mice
Source: Front Immunol. 2020 Jul 29;11:1652. doi: 10.3389/fimmu.2020.01652 (PMC7403191; doi:10.3389/fimmu.2020.01652)
Supplement: Supplementary file 3 [file Table_3.PDF]

Supplemental Table S3. Features of the 12 hr Model (Males vs. Females).

Feature	p-value	Coefficient
Neutrophils_pSTAT3	0.000575895	-0.209634985
Tregs_pStat6	0.000987248	0.029068354
Tregs_pSTAT1	0.001563143	0.006234159
CD49+NK11+NK_pStat6	0.005512135	0.002153976
CD8Tmem_pSTAT1	0.005512135	0.00660584
CD19+IgM-B_pSTAT3	0.007897984	-0.045675759
CD4T_p-p38	0.007897984	-0.004689443
pDCs_pNFkB	0.007897984	-0.004367682
CD49+NK11+NK_pSTAT5	0.007897984	2.68E-05
gdT	0.011720404	-0.017791788
Neutrophils	0.020649938	0.009662821
Tregs_pNFkB	0.020649938	0.018015711
CD19+IgM-B_pCREB	0.027396133	-0.001721447
intMCs_pSTAT3	0.035952283	-0.000553629
CD49+NK11+NK_pSTAT1	0.035952283	0.003868141
CD49+NK11+NK_pCREB	0.035952283	0.033558906
CD4T_pS6	0.046400658	-0.000696778
NKT_pSTAT5	0.046400658	0.000361198
ncMCs_pS6	0.059234883	-0.03912425
intMCs_pS6	0.059234883	-0.002542297
CD4T_pMAPKAPK2	0.074454957	-0.006503651
CD4Tmem	0.074870783	-0.054977952
CD19-IgM-B_pMAPKAPK2	0.092719046	0.000141189
Bcells_pNFkB	0.113944879	-0.005813559
CD4Tnaive_pSTAT5	0.113944879	0.043019273
CD4Tnaive_p-p38	0.138790621	-0.021687277
CD4Tmem_CD62L	0.138790621	0.012748619
Tregs_pERK	0.138790621	0.013545131
intMCs_pStat6	0.138790621	0.014845567
mDCs_pS6	0.167174002	-0.021383835
CD19+IgM-B_pNFkB	0.167174002	-0.003208644
CD19+IgM+B_pNFkB	0.167174002	-0.000346941

CD8T_pSTAT5	0.167174002	0.002870799
Tregs_pSTAT5	0.199588647	0.000836279
ncMCs_pNFkB	0.235870012	-0.001287516
gdT_pSTAT1	0.235870012	1.82E-05
gdT_pStat6	0.235870012	0.001867313
pDCs_pSTAT5	0.235870012	0.002151443
ncMCs_pSTAT5	0.276593994	0.010857121
cMCs	0.289543008	-0.002127832
ncMCs_pERK	0.321266968	-0.003754416
CD8Tmem_CD62L	0.321266968	0.001137308
intMCs_pSTAT5	0.370382559	0.001294447
CD8Tnaive_pSTAT5	0.370382559	0.001763187
cMCs_pSTAT5	0.370382559	0.023109004
CD49+NK11+NK	0.384722989	-0.004967741
CD19-IgM-B_pCREB	0.423447141	0.007151582
ncMCs_CD62L	0.480707528	-0.022527293
CD4T_pERK	0.480707528	-0.000870364
NKT_CD62L	0.541423283	0.00070358
CD19-IgM-B_pSTAT1	0.541423283	0.001523332
Neutrophils_pStat6	0.541423283	0.001689719
intMCs_pCREB	0.541423283	0.001800366
CD49+NK11-NK_CD62L	0.541423283	0.00197569
CD19+IgM-B_CD62L	0.605841218	-0.001267307
CD19-IgM-B_pSTAT5	0.605841218	0.018660872
mDCs_pSTAT1	0.672974085	0.000909319
mDCs_pMAPKAPK2	0.672974085	0.00108436
gdT_pERK	0.742986425	-0.000448199
CD8Tnaive_pERK	0.742986425	-0.00027054
Tregs_pMAPKAPK2	0.814808721	0.018855473
Tregs_pS6	0.962566845	0.000183836
cMCs_pStat6	0.962566845	0.000376597
intMCs_pERK	1	0.000148734
mDCs	1	0.000304015
