# Supplementary material for: Systematic Immunophenotyping Reveals Sex-Specific Responses After Painful Injury in Mice
Source: Front Immunol. 2020 Jul 29;11:1652. doi: 10.3389/fimmu.2020.01652 (PMC7403191; doi:10.3389/fimmu.2020.01652)
Supplement: Supplementary file 4 [file Table_4.PDF]

Supplemental Table S4. Features of the 7 Day Model (Males vs. Females)

Feature	p-value	Coefficient
CD4Tmem_p-p38	0.0001554	-0.002063077
pDCs_pCREB	0.0001554	-0.002361383
CD4Tmem_pERK	0.0001554	-0.108933164
CD19+IgM+B_CD62L	0.000621601	-0.024117698
cMCs	0.000621601	-0.038030371
CD19-IgM-B	0.001087801	-0.002900917
pDCs_p-p38	0.001087801	-0.018820384
CD4Tmem_pNFkB	0.001087801	-0.030154287
CD19+IgM-B_CD62L	0.001087801	-0.077235968
CD4Tmem_pMAPKAPK2	0.001087801	-0.113517802
pDCs_pMAPKAPK2	0.001864802	-0.020908603
mDCs_p-p38	0.002952603	-0.006323731
CD19-IgM-B_pCREB	0.006993007	-0.000206239
CD19+IgM+B	0.01041181	0.002095558
CD4Tmem_CD62L	0.014763015	0.003257027
CD19-IgM-B_p-p38	0.028127428	-0.000413348
Neutrophils_pSTAT3	0.037917638	-0.01743663
Neutrophils_pNFkB	0.04988345	0.001006339
intMCs_pCREB	0.04988345	-0.003460887
CD19-IgM-B_pMAPKAPK2	0.04988345	-0.021692609
intMCs_pERK	0.064957265	-0.000408527
mDCs_pSTAT3	0.064957265	-0.001036695
NKT_pSTAT3	0.064957265	-0.004167777
CD49+NK11-NK_CD62L	0.082983683	-0.025183045
Neutrophils_IkB	0.104895105	1.17E-05
CD19+IgM-B	0.13038073	-0.014593982
pDCs_pNFkB	0.160528361	-0.000113069
CD49+NK11+NK_pSTAT3	0.194871795	-0.001708863
mDCs_pStat6	0.234498834	0.029862721

mDCs_pERK	0.234498834	-0.000662805
Neutrophils_pStat6	0.234498834	-0.00189813
Tregs_pCREB	0.278632479	0.000895249
mDCs_pSTAT1	0.278632479	0.000250629
CD19-IgM-B_pERK	0.278632479	-0.000263927
intMCs_CD62L	0.328205128	0.02637797
pDCs_pStat6	0.328205128	-0.022774089
CD8T_pSTAT1	0.382284382	0.007084423
CD8Tnaive_pSTAT1	0.382284382	0.006378636
NKT_pS6	0.382284382	0.000920123
CD4Tnaive_pMAPKAPK2	0.382284382	-7.33E-05
CD8T_pSTAT5	0.441802642	0.00376361
CD8Tnaive_pSTAT5	0.441802642	0.001331191
ncMCs_CD62L	0.441802642	0.000903966
mDCs	0.560041003	0.050842007
pDCs_IkB	0.573737374	0.00131485
NKT_pStat6	0.573737374	-0.001273032
CD49+NK11+NK_pSTAT5	0.645376845	-0.001559655
NKT_CD62L	0.720901321	-0.001337705
CD8T_pNFkB	0.878477078	0.000590954
CD49+NK11-NK_pCREB	0.959129759	0.000847755
CD49+NK11+NK_IkB	0.959129759	0.000340646
CD49+NK11+NK_pStat6	0.959129759	-0.00504225
