# Supplementary material for: Systematic Immunophenotyping Reveals Sex-Specific Responses After Painful Injury in Mice
Source: Front Immunol. 2020 Jul 29;11:1652. doi: 10.3389/fimmu.2020.01652 (PMC7403191; doi:10.3389/fimmu.2020.01652)
Supplement: Supplementary file 5 [file Image_1.PDF]

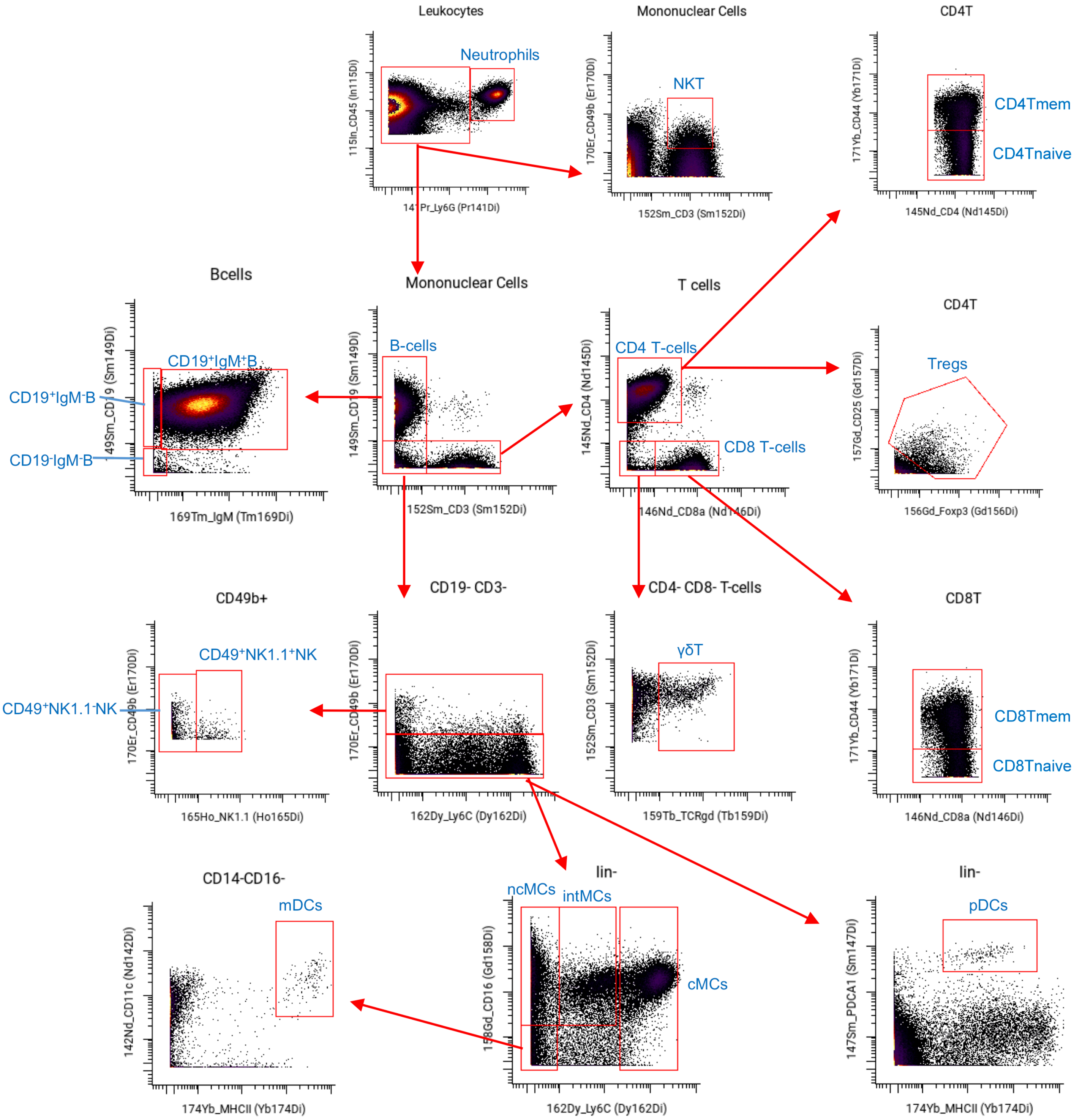

Leukocytes
Neutrophils
115In_CD45 (In115Di)
141Pr_Ly6G (Pr141Di)
Mononuclear Cells
NKT
170Er_CD49b (Er170Di)
152Sm_CD3 (Sm152Di)
CD4T
CD4Tmem
CD4Tnaive
171Yb_CD44 (Yb171Di)
145Nd_CD4 (Nd145Di)
Bcells
CD19+IgM+B
CD19+IgM-B
CD19-IgM-B
149Sm_CD19 (Sm149Di)
169Tm_IgM (Tm169Di)
Mononuclear Cells
B-cells
149Sm_CD19 (Sm149Di)
152Sm_CD3 (Sm152Di)
T cells
CD4 T-cells
CD8 T-cells
145Nd_CD4 (Nd145Di)
146Nd_CD8a (Nd146Di)
CD4T
Tregs
157Gd_CD25 (Gd157Di)
156Gd_Foxp3 (Gd156Di)
CD49b+
CD49+NK1.1+NK
CD49+NK1.1-NK
170Er_CD49b (Er170Di)
165Ho_NK1.1 (Ho165Di)
CD19- CD3-
170Er_CD49b (Er170Di)
162Dy_Ly6C (Dy162Di)
CD4- CD8- T-cells
γδT
152Sm_CD3 (Sm152Di)
159Tb_TCRgd (Tb159Di)
CD8T
CD8Tmem
CD8Tnaive
171Yb_CD44 (Yb171Di)
146Nd_CD8a (Nd146Di)
CD14-CD16-
mDCs
142Nd_CD11c (Nd142Di)
174Yb_MHCII (Yb174Di)
lin-
ncMCs
intMCs
cMCs
158Gd_CD16 (Gd158Di)
162Dy_Ly6C (Dy162Di)
lin-
pDCs
147Sm_PDCA1 (Sm147Di)
174Yb_MHCII (Yb174Di)

Supplemental Figure S1. **Gating strategy.** Two-dimensional dot plots are shown for a representative mouse sample. Gating was performed using Cytobank (www.cytobank.org). Twenty-one innate and adaptive cell types were manually gated and included in the analysis.

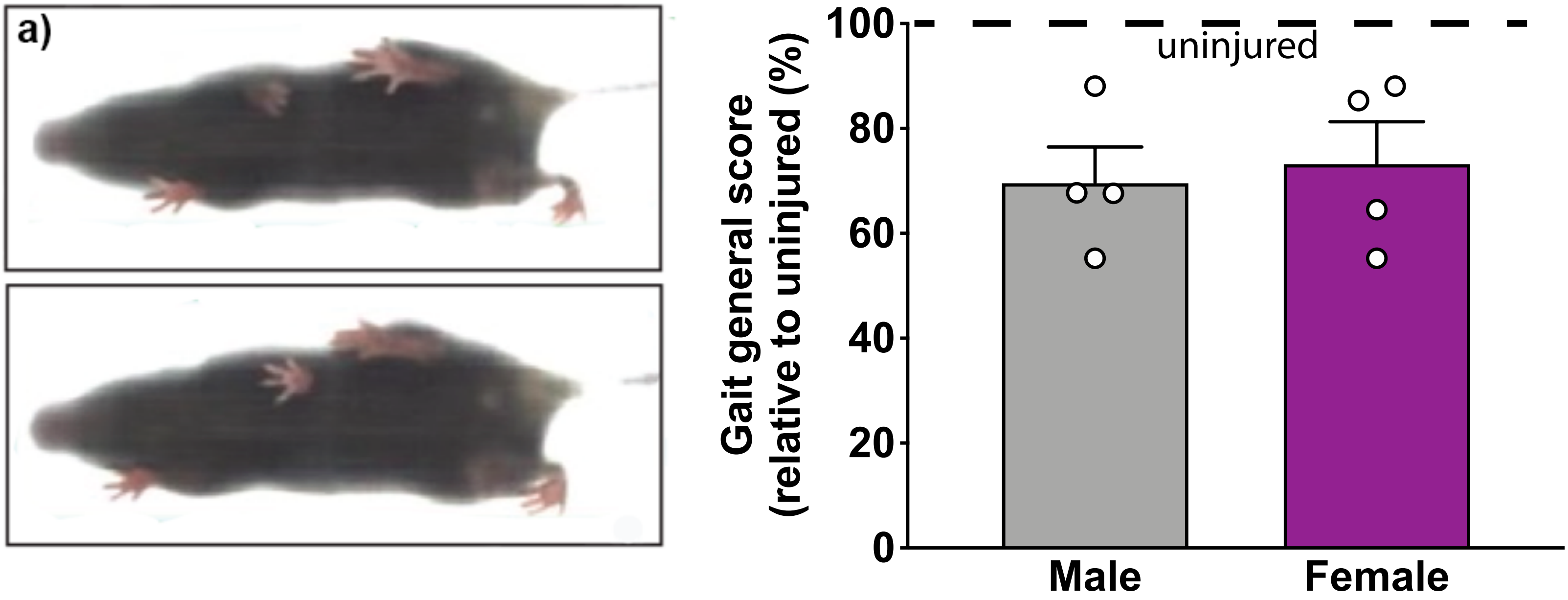

Supplemental Figure S2. Mouse gait analysis reveals altered dynamics after injury. a) Representative image from DigiGait system in which mice (n = 8 per group) were positioned on a transparent treadmill and a video camera was placed underneath to record the gait while taking strides on 15 cm/s belt.
b) Quantification of the gait ‘disability score’ at Day 7 resulting from the analysis of 42 parameters (see Methods). Group mean for each limb + SEM is shown.

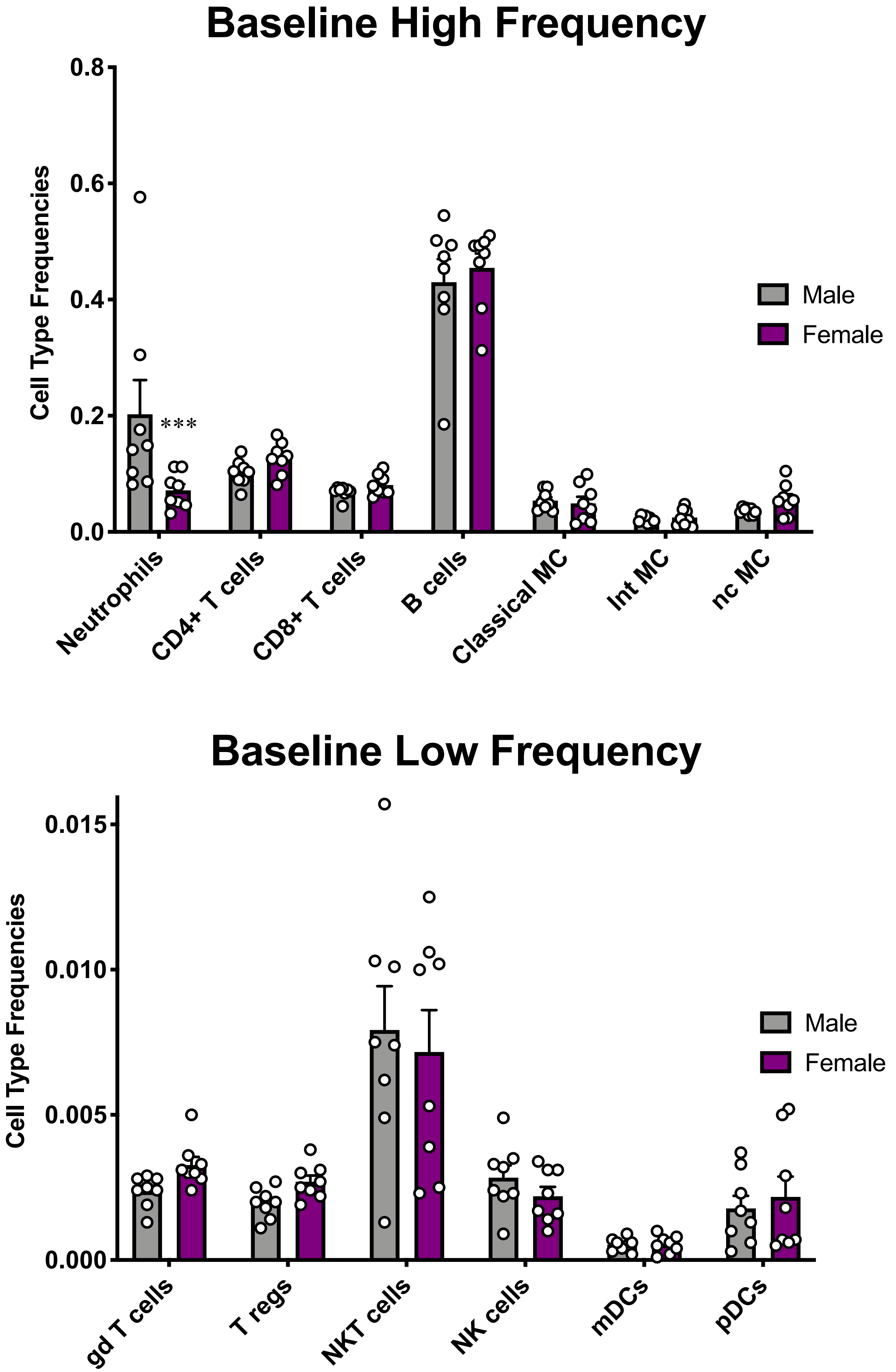

Supplemental Figure S3. Manual gating for immune cell populations reveals sex-conserved frequencies at baseline, except for neutrophils. The thirteen most highly expressed cell types are shown with baseline frequencies by manual gating. Neutrophils were more highly expressed in males (****p*<0.001 vs. females, by unpaired t-test). Note change in y-axis between high frequency and low frequency populations.

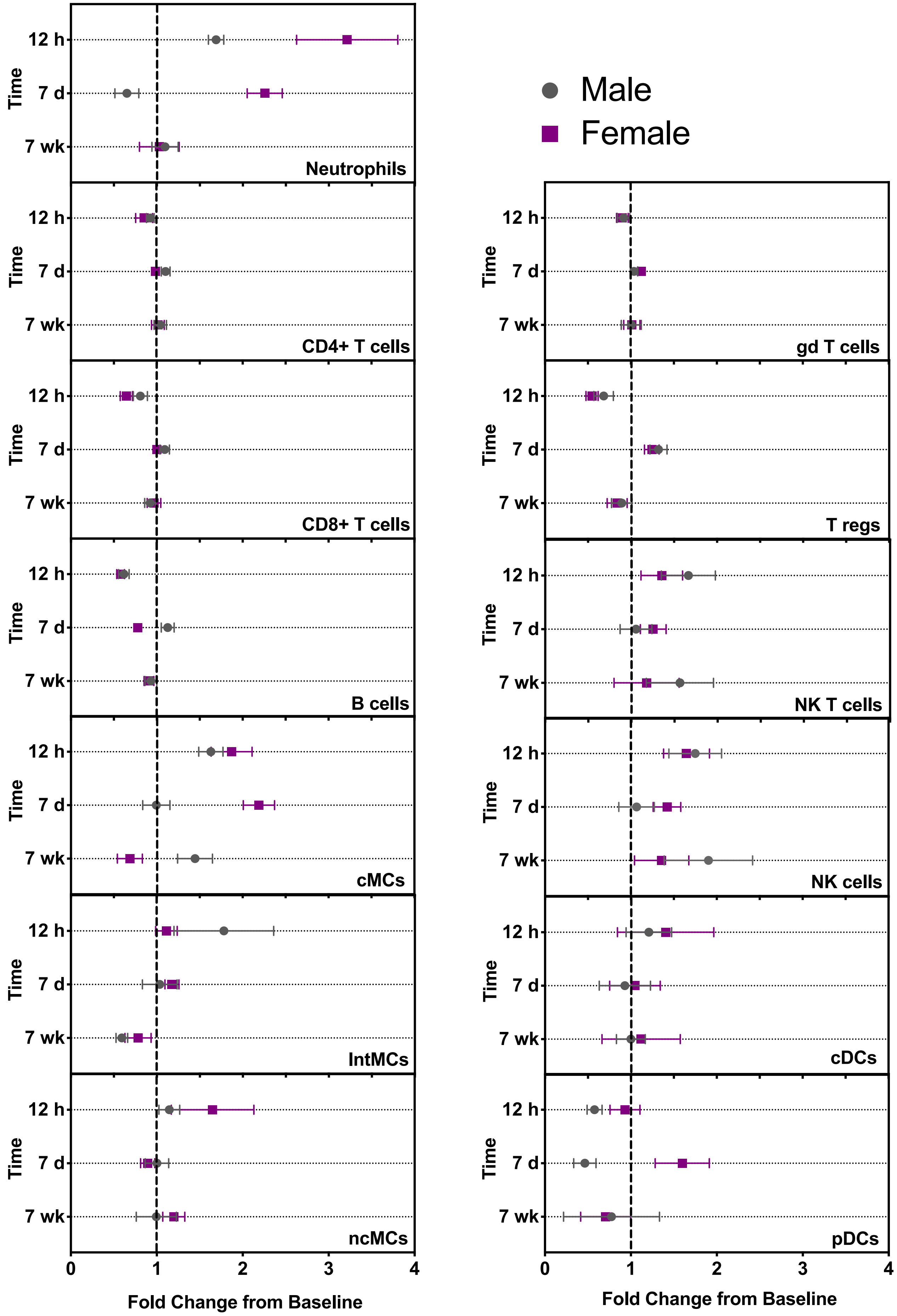

Supplemental Figure S4. Change in cell frequencies reveals expected early expansion of neutrophils in both sexes. Fold change from baseline is shown for the thirteen most highly expressed cell types by manual gating.

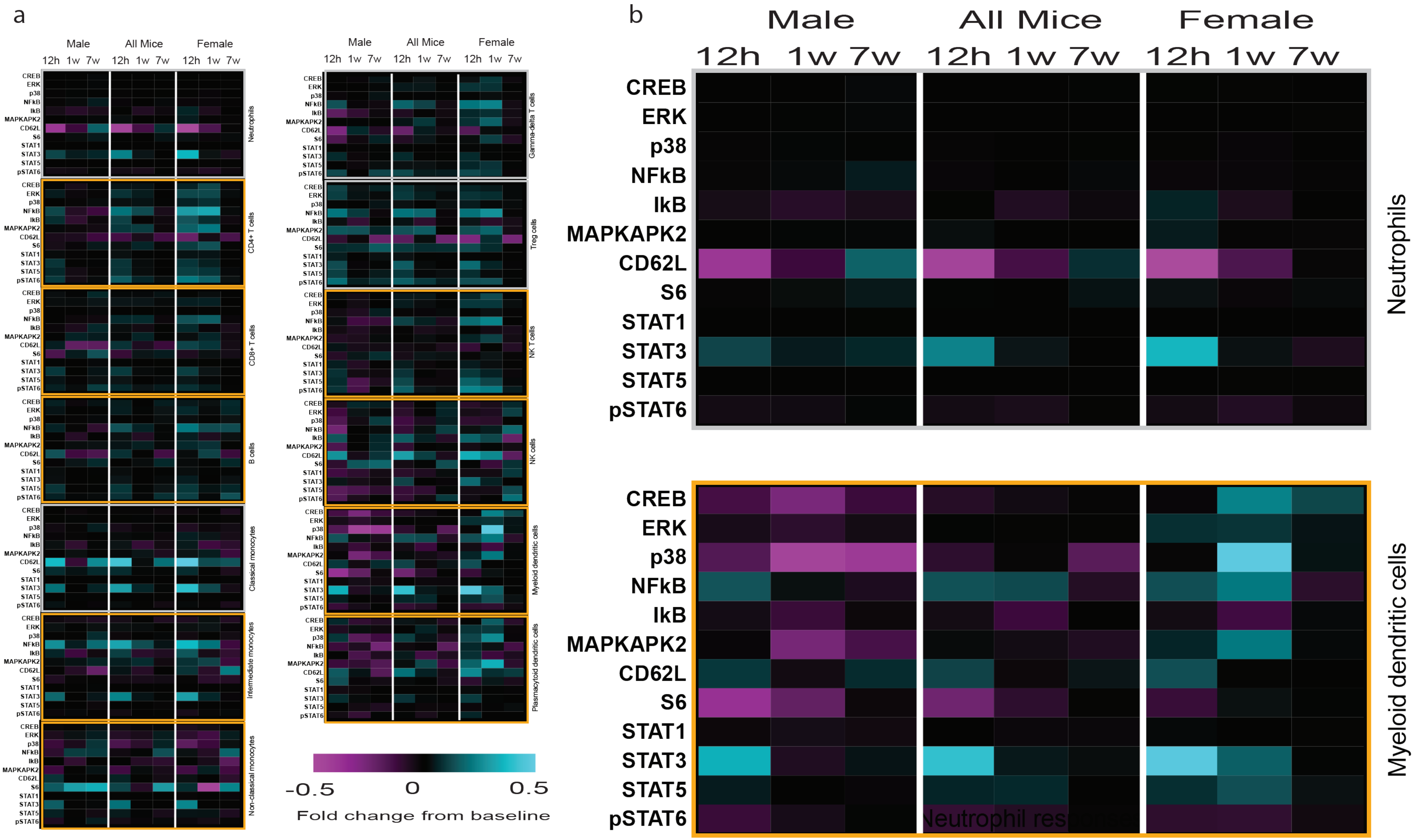

Supplemental Figure S5. Combining data from both sexes masks sex-divergent immune responses to injury.
a) Thirteen representative cell types and their intracellular signaling responses over time after injury are shown as a heat map of fold-change from baseline. Cell types in which data from both sexes is visually similar are boxed in gray while those that suggest sex-divergence are boxed in orange. Left column: Males; Center column: Combined sexes; Right column: Females.
b) Enlarged version of two cell types demonstrates that some features respond in a sex-independent manner to injury (top panel: neutrophils) while others demonstrate a sex-specific response (bottom panel: myeloid dendritic cells).

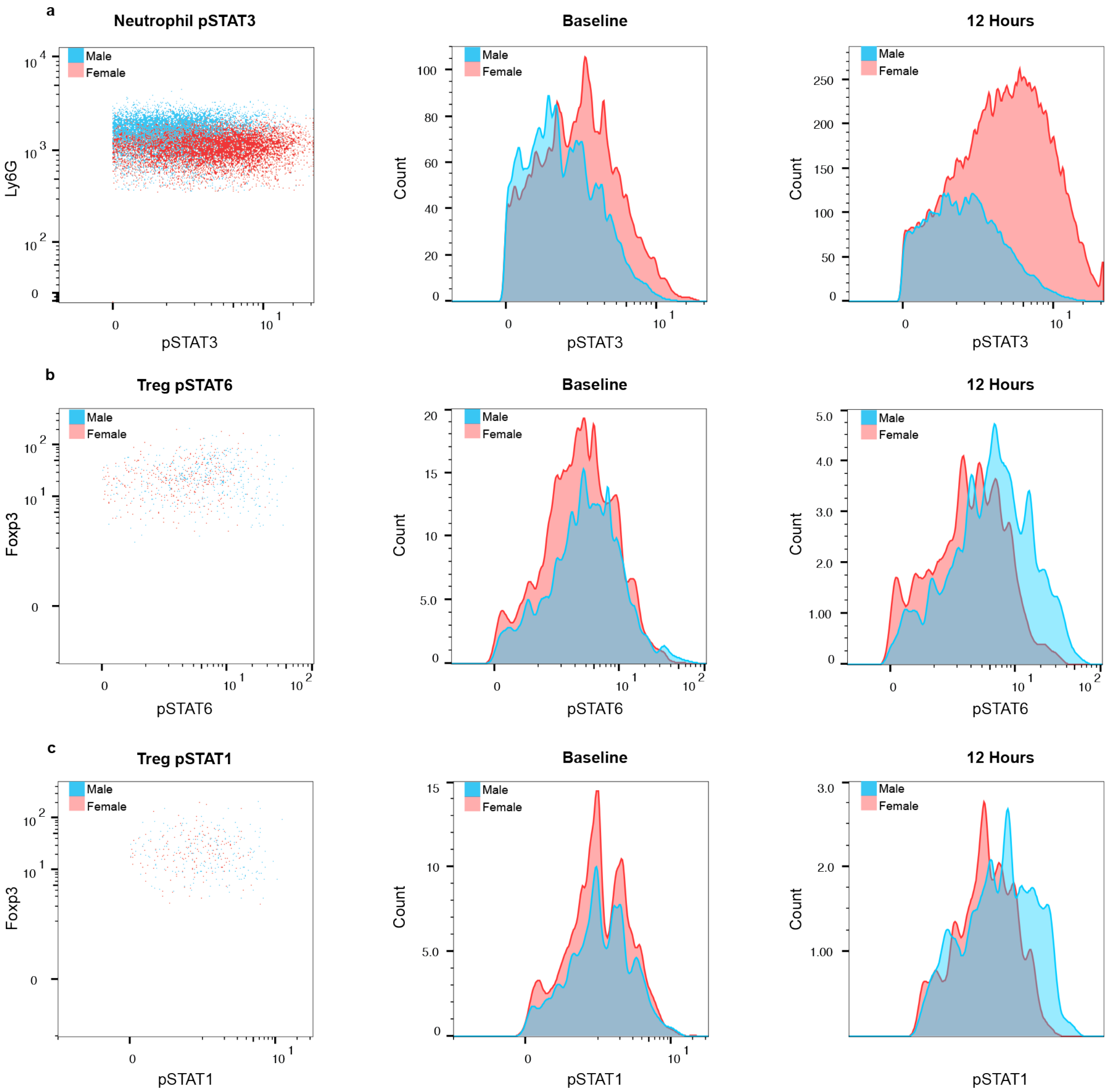

Supplemental Figure S6. Representative FACS plots and histograms for most highly informative components of the early (immediate postoperative) model. Manually gated populations are shown compared to intracellular signaling marker in left plots. Histograms demonstrate count (y-axis) vs. intracellular signaling expression (x-axis). Right shift represents change in median expression for a) neutrophil pSTAT3, b) Treg pSTAT6 and c) Treg pSTAT1.

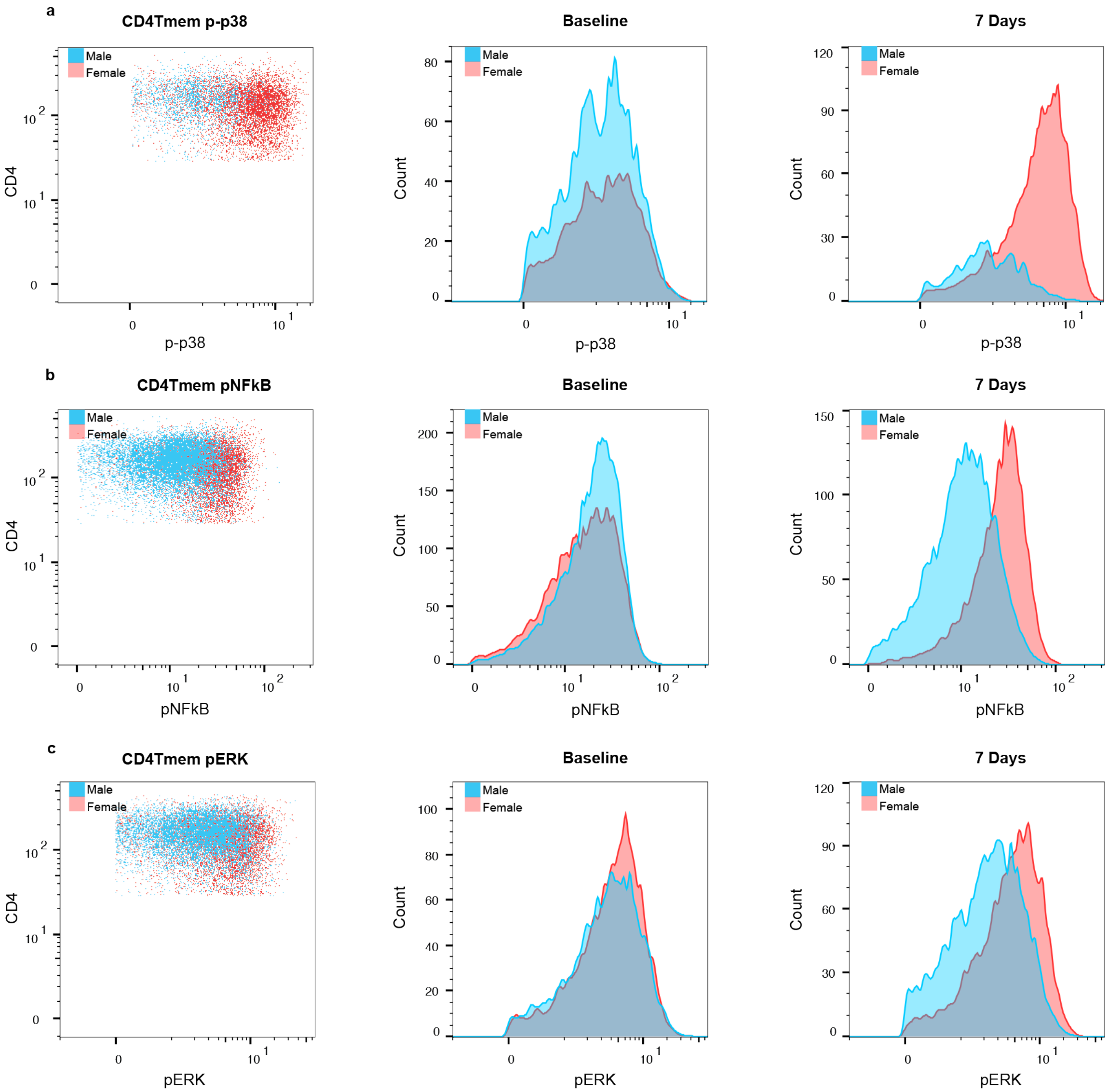

Supplemental Figure S7. Representative FACS plots and histograms for most highly informative components of the late (subacute) model. Manually gated populations are shown compared to intracellular signaling marker in left plots. Histograms demonstrate count (y-axis) vs. intracellular signaling expression (x-axis). Right shift represents change in median expression for a) CD4Tmem p-p38, b) CD4Tmem pNFkB and c) CD4Tmem pERK.

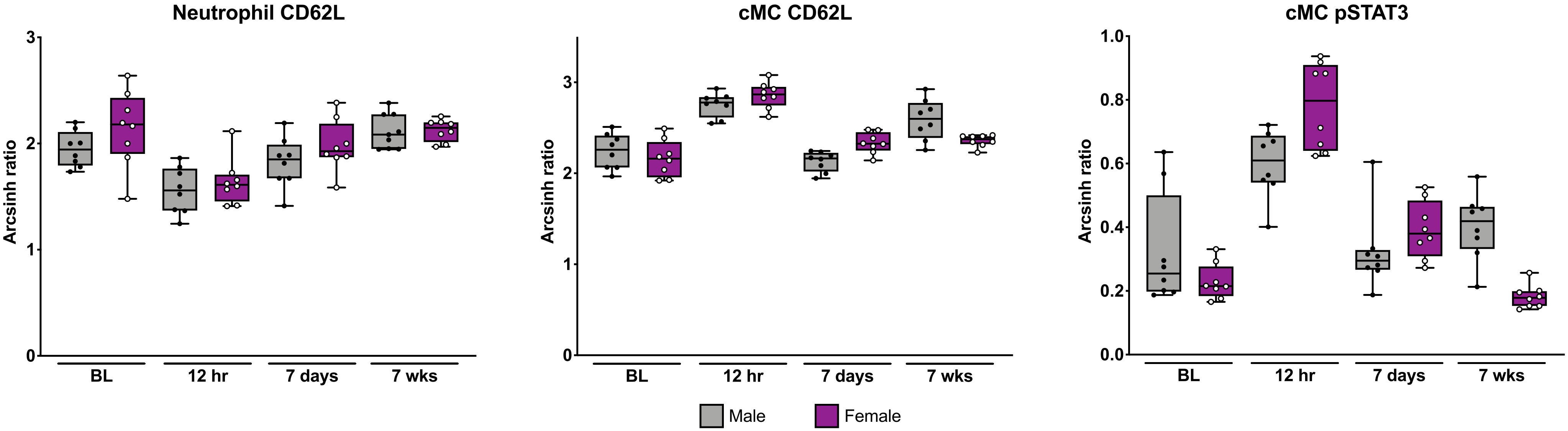

Supplemental Figure S8. Example features that change after injury and are conserved between sexes. We highlight 3 features that are changed in similar fashion after injury in both males and females. There are several features that were conserved between sexes including canonical injury responses. Specifically we observed 1) a decrease in neutrophil CD62L at 12 hours in both sexes suggesting rapid egress of neutrophils from blood to the tissue site of injury; 2) an increase in classical monocyte CD62L at 12 hours and then subsequent decrease at 7 days suggesting a delayed transmigration into tissues and 3) an increase in classical monocyte pSTAT3 at 12 hours suggesting immediate activation of these cells after injury.

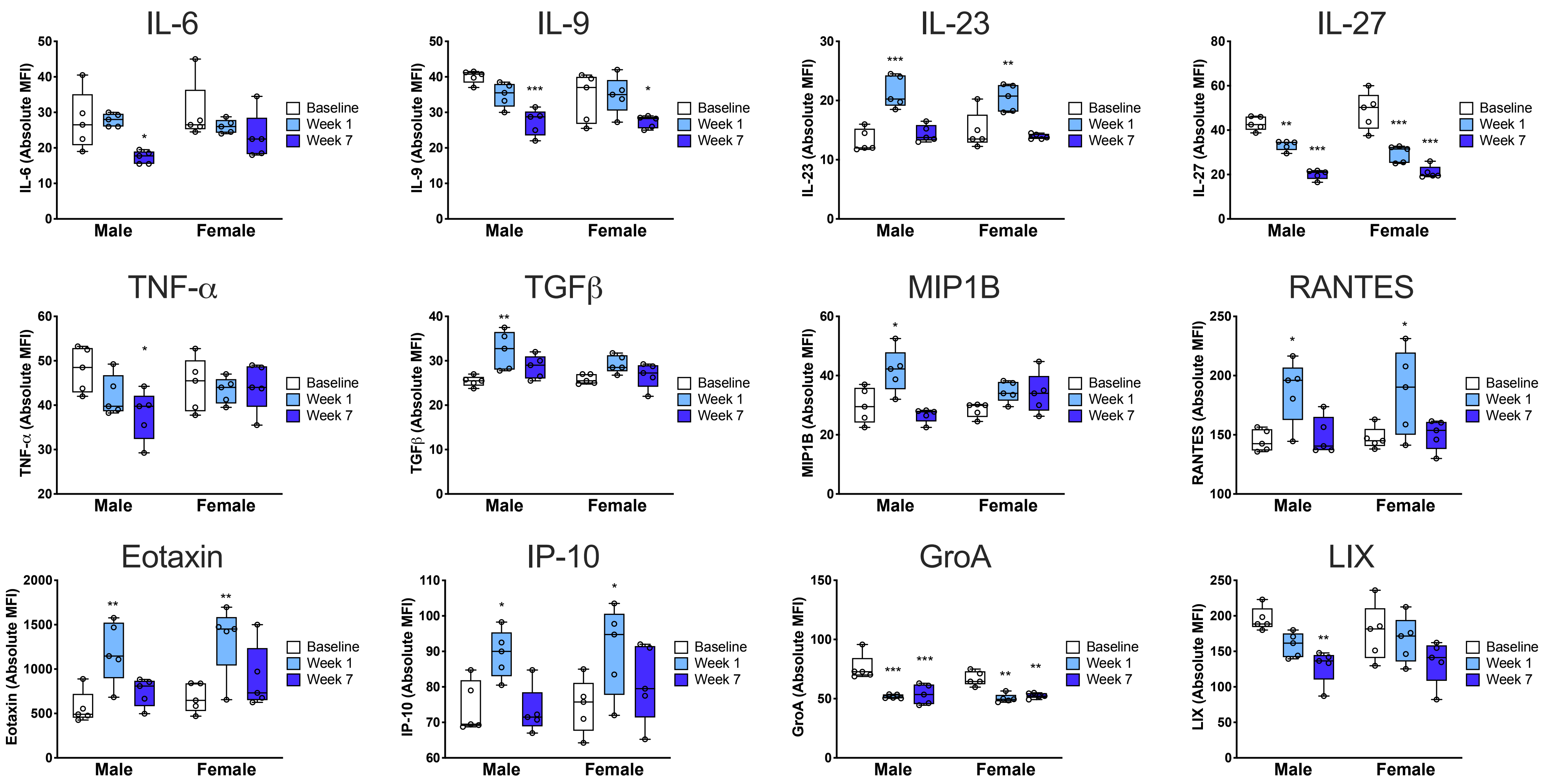

Supplemental Figure S9. Cytokine profile from the injured limb demonstrates sex similarities in the tissue immune response. Paw skin protein was subjected to multiplex enzyme-linked immunosorbent assay to determine the levels of key cytokines at baseline, 7 days and 7 weeks after injury. We identified twelve proteins with changes that were either sex-conserved or only changed in one sex. Males and females both exhibited increased levels of IL-23, eotaxin, RANTES, and IP-10 at 7 days and decreased levels of IL-9, IL-27 and GroA at 7 days and 7 weeks. (n = 5 mice per group per sex, $^{*}p < 0.05$, $^{**}p < 0.01$, $^{***}p < 0.001$ vs. respective sex baseline, by two-way ANOVA, Sidak's post-test).
